# Stability of yield and its components in grafted tomato tested across multiple environments in Texas

**DOI:** 10.1038/s41598-020-70548-3

**Published:** 2020-08-11

**Authors:** Desire Djidonou, Daniel I. Leskovar, Madhumita Joshi, John Jifon, Carlos A. Avila, Joseph Masabni, Russell W. Wallace, Kevin Crosby

**Affiliations:** 1 Texas A&M AgriLife Research, Texas A&M University, Uvalde, TX 78801 USA; 2 Texas A&M AgriLife Research, Texas A&M University, Weslaco, TX 78596 USA; 3 Texas A&M AgriLife Extension, Texas A&M University, Overton, TX 7568 USA; 4 Texas A&M AgriLife Extension, Texas A&M University, Lubbock, TX 79403 USA; 5 Department of Horticultural Sciences, Texas A&M University, College Station, TX 77845 USA

**Keywords:** Genetics, Plant sciences, Environmental sciences

## Abstract

Grafting with vigorous rootstocks could offer tomato growers in Texas sustainable and efficient option to achieve reliable yield across a range of production systems and locations. Genotypes (G) of grafted and non-grafted tomato were grown in different environments (E) in the 2017 and 2018 spring seasons. The objectives of the study were to (i) evaluate the effects of production system and grafting on tomato yield traits, (ii) determine the size of genotypic and genotype by environment interaction (G × E) variance components, and (iii) evaluate the relative stability of tested genotypes for yield and its components across production environments. In 2017, genotypes were non-grafted ‘TAMU Hot Ty’ (TAM) and ‘Tycoon’ (TY) and each grafted on commercial tomato rootstocks ‘Estamino’ (TAM/ES, TY/ES) and ‘Multifort’ (TAM/MU, TY/MU) while in 2018, TAM and ‘HM1823’ (HM) were grafted on ‘Estamino’ (TAM/ES, HM/ES) and ‘Multifort’ (TAM/MU, HM/MU). Testing environments were high tunnel (HT) and open-field (OF) in Uvalde in 2017 while in 2018, these were HT and OF in Lubbock (LU-HT, LU-OF), Overton (OV-HT, OV-OF), Uvalde (UV-HT, UV-OF), and Weslaco (WE-HT, WE-OF). Total and marketable yields, fruit number per plant, and average fruit weight were significantly affected by E, G, and G × E interaction. Environmental component contributed 71–86% to the total variation for all these traits, while genotype explained 1.5–10.8%, and the contribution of G × E ranged between 4.3 to 6.7%. Estimation of the univariate statistic parameters and genotype plus genotype × environment (GGE) biplot analysis indicated that HM/MU and HM/ES were the most stable graft combination with the highest total and marketable yields, while TAM/ES was very unstable for yields across test environments. TAM/MU was stable but with yield lower than the grand mean. These results suggest that high tomato yields could be consistently achieved with grafted combination (HM/MU and HM/ES) especially under high tunnel production system across the regions of Texas.

## Introduction

Genetics, environmental factors, and cultural management practices typically determine the extent of quantitative traits of economic importance such as genotype or cultivar yield. As such, the proportion of the variation in this phenotypic trait due to the main effect of genotype (G), the environment (E) and their interactions (G × E) is routinely assessed during the process of selecting top-performing lines^1^. In this regard, multilocation trials are routinely conducted to assess the pattern of genotypic response over a number of locations and years (test environments). Typically, a significant G × E exists whenever the genotype performance greatly differs with environments^2,3^. When a significant G × E is identified, subsequent stability analysis is often carried out to identify stable genotypes which exhibit consistently higher yield over several environments^4^. The presence of significant G × E has been found in growth and yield traits of several field^5–8^ and vegetable crops^3,9–11^. In tomato (*Solanum lycopersicum* L.), an economically important vegetable widely grown and consumed in the world, yield and quality traits were found to demonstrate strong G × E^12–16^. Concurrent stability studies in yield traits have allowed the separation of population of tomato varieties into highly stable and highly unstable^17–20^.

These stability analyses have been focused on hybrid cultivars and breeding lines of tomatoes but not so on composite hybrid plants that are created through the grafting technique. Generally viewed as an alternative to conventional breeding of elite cultivars, grafting of vegetable seedlings combines desirable traits from the scion and rootstock in a single composite plant^21^. For tomato and other high market-value vegetable crops, such desirable traits imparted by the rootstock include resistance to multiple soil-borne pathogens, tolerance to abiotic stresses and enhanced vigor^22,23^. As a result, grafted tomato plants often exhibit improved overall health and vigor even under contrasting environmental conditions, leading to stable or enhanced yields. Bie et al.^24^ reported increased yields of grafted tomato ranging from 5.4 to 80.3%. Reported variation in yields of grafted tomato plants is primarily related to specific scion/rootstock combinations and the environmental variations in specific production systems.

A recent meta-analysis of 949 combinations of scion/rootstock of different cultivars in tomatoes performance found that grafted plant yields were not significantly higher in 65% of the combinations, although an average 37% yield increase was observed over all data evaluated^25^. These authors also found that grafted/non-grafted yield ratios in 105 grafted treatments with rootstock ‘Maxifort’ varied widely with specific scion. In addition to variation in scion/rootstock combination, the relative performance of grafted plants also greatly varies with different growing environments, especially protected environment (greenhouse, high tunnel) or open-field system.

However, while there are several studies that evaluated the growth and yield performance of grafted tomatoes in protected structure^26–29^ and open field^30–32^, few examined the performance of different scion/rootstock combinations simultaneously in the protected environment (high tunnel) compared to the open‐field system across different locations. These observations indicate that limited information is available on the stability of yield traits of specific scion/rootstock combinations of tomato grown across a range of environments in general and Texas in particular.

Efficient production of fresh-market tomato in Texas has long been limited by a number of challenges including biotic and abiotic stresses (especially episodes of high temperature and drought), inadequate cultivars and production practices^33^. With the goal to revitalize the industry given in part the increasing consumer demand for locally-grown vegetables, the Texas A&M AgriLife Research breeding programs have recently intensified the development of improved cultivars with potential for heat tolerance, fruit quality, and disease resistance^33^. In addition, vegetable grafting could complement these efforts in ensuring an effective management of biotic and abiotic stresses and enhancing tomato plant growth and productivity. However, it is currently unclear whether specific scion/rootstock combinations consistently exhibit stable and improved yields irrespective of the production system (high tunnel or open field) within or across locations. There is a need for in-depth G × E studies of grafted tomato combinations across a range of environments (production systems, locations, and years) to demonstrate genotypic stability of economically important traits. The objectives of this study were to (i) evaluate the effects of production system and grafting on tomato yield traits, (ii) determine the size of genotypic and genotype by environment interaction (G × E) variance components, and (iii) evaluate the relative stability of tested genotypes for yield and its components across production environments.

## Material and methods

### Plant material and testing environments

The experiments were initiated during the spring seasons of 2017 and 2018. Different sets of grafted treatments were included in each trial in the 2017 and 2018 spring seasons. In 2017 season, two determinate cultivars ‘TAMU Hot Ty’ (TAM) and ‘Tycoon’ (TY) were grafted each on ‘Estamino’ (TAM/ES, TY/ES) and ‘Multifort’ (TAM/MU, TY/MU). Due to discontinuity of Tycoon seed in the market, TY was replaced by cultivar ‘HM1823′ in the 2018 season. The six grafted treatments in 2018 season included non-grafted ‘TAMU Hot Ty’ (TAM) and ‘HM1823′ (HM) and grafted on ‘Estamino’ (TAM/ES, HM/ES) and ‘Multifort’ (TAM/MU, HM/MU). Grafted and non-grafted referred as genotypes (G) were tested in two environments including high tunnel (HT) and open-field (OF) in Uvalde (UV-HT, UV-OF) during the 2017 season and in eight environments which included HT and OF in Lubbock (LU-HT, LU-OF), Overton (OV-HT, OV-OF), Uvalde (UV-HT, UV-OF), and Weslaco (WE-HT, WE-OF) during the 2018 season (Table 1).

**Table 1:** Environments description, geographic coordinates of test locations, and cultural practices for the evaluation of grafted and non-grafted tomato in Texas.

Location	Production system	Env	Altitude	Latitude	Longitude	Soil type	Date of transplanting	Date of final harvest	Number of harvests
			m	^o^S	^o^W				
Lubbock	HT	LU-HT	991	33	101	Olton loam	5/1/2018	10/4/2018	14
	OF	LU-OF					5/11/2018	9/11/2018	6
Overton	HT	OV-HT	153	32	94	Sandy loam	3/23/2018	7/20/2018	7
	OF	OV-OF					4/5/2018	7/20/2018	5
Uvalde	HT	UV-HT	276	29	99	Silty clay loam	2/1/2018	7/3/2018	10
	OF	UV-OF					3/26/2018	7/2/2018	5
Weslaco	HT	WE-HT	23	26	97	Sandy loam	3/2/2018	6/25/2018	6
	OF	WE-OF					3/2/2018	6/25/2018	6

HT: high tunnel, OF: open-field, Env.: environments.

### Experimental design and cultural practices

At each location and production system, the six grafted treatments (genotypes) were arranged in a randomized complete block design with three to five replications (block).

Planting dates varied with locations and with production system within each location (Table 1). Across all testing environments, plants were grown on raised beds covered with plastic mulch and installed with a single drip tape (per row) for subsurface drip fertigation. Beds were spaced 1.52 m apart (center to center) with 0.60 m in-row spacing for open-field tomato production, resulting in a planting density of 11,960 plants/ha. Depending on location and production system, experimental unit consisted of 5 to 10 plants. Other cultural management practices for tomato production, including irrigation, fertilization application and disease/insect control were carried out routinely according to commercial production guidelines specific to each location^34^.

### Data collection

Date of start of harvest and number of harvests varied with environments (Table 1). In each environment, fruit were harvested at pink to red ripeness stages from 5 to 10 plants in each grafted treatment per replication. Fruit was graded as extra-large, large, medium (marketable categories), and culls (small and defective fruit). Fruits in each grade were counted and weighed. Total and marketable fruit yield (Mg ha^−1^), average fruit weight (g fruit^−1^), and average number of fruit per plant (no. plant^−1^) were estimated.

### Data analysis

Due to differential treatment structures in the two growing seasons, data were analyzed separately. In 2017, a two-way analysis of variance (ANOVA) was performed for yield and yield components using a generalized linear mixed model procedure (SAS 9.4 version, SAS Inst., Cary, N.C., USA). When significant effects were observed, means separation was performed to examine differences between production systems (HT vs OF) and among the grafted treatments using Tukey’s test (*P* ≤ 0.05).

In 2018, yield data were subjected to a combined ANOVA for genotype, environment, and G × E interaction with location, production system, replications, and genotypes as random effects. The percentages of G, E, and G × E sum of squares of the total variation were estimated to evaluate the magnitude of variation contributed by each factor. Least squared means and significance (based on Tukey’s adjustment for multiple mean comparisons) were computed for each trait for each genotype and environment. When significant G × E was detected for a specific response, stability analysis was carried out with estimation of univariate stability indexes and multivariate stability statistics based on genotype plus genotype by environment interaction approach (GGE biplot). The SASG x E program developed by Dia et al.^35^ was used to compute the univariate stability coefficients including the regression slope (${b}_{i}$), deviation from regression (${S}_{d}^{2}$), and Shukla’s stability variance (${\sigma }_{i}^{2}$). Also, the GGE biplots analysis was carried out using the ‘GGEBiplotGUI’ package of R statistical software^36,37^. This graphical analysis mainly involved (i) mega-environment analysis (“which-won-where” pattern of GGE biplot), (ii) genotype evaluation (mean vs. stability analysis, genotypes ranking with “ideal” genotype), and (iii) testing environment evaluation (especially, discriminating power of environments, ranking of genotypes in specific environments).

### Disclaimer

Mention of a variety name, trademark, or vendor does not constitute a guarantee or warranty of the product by the authors and does not imply its approval to the exclusion of other products or vendors that may also be suitable.

## Results

### Microclimate under high tunnel and open field in Uvalde location

**Figure 1:**
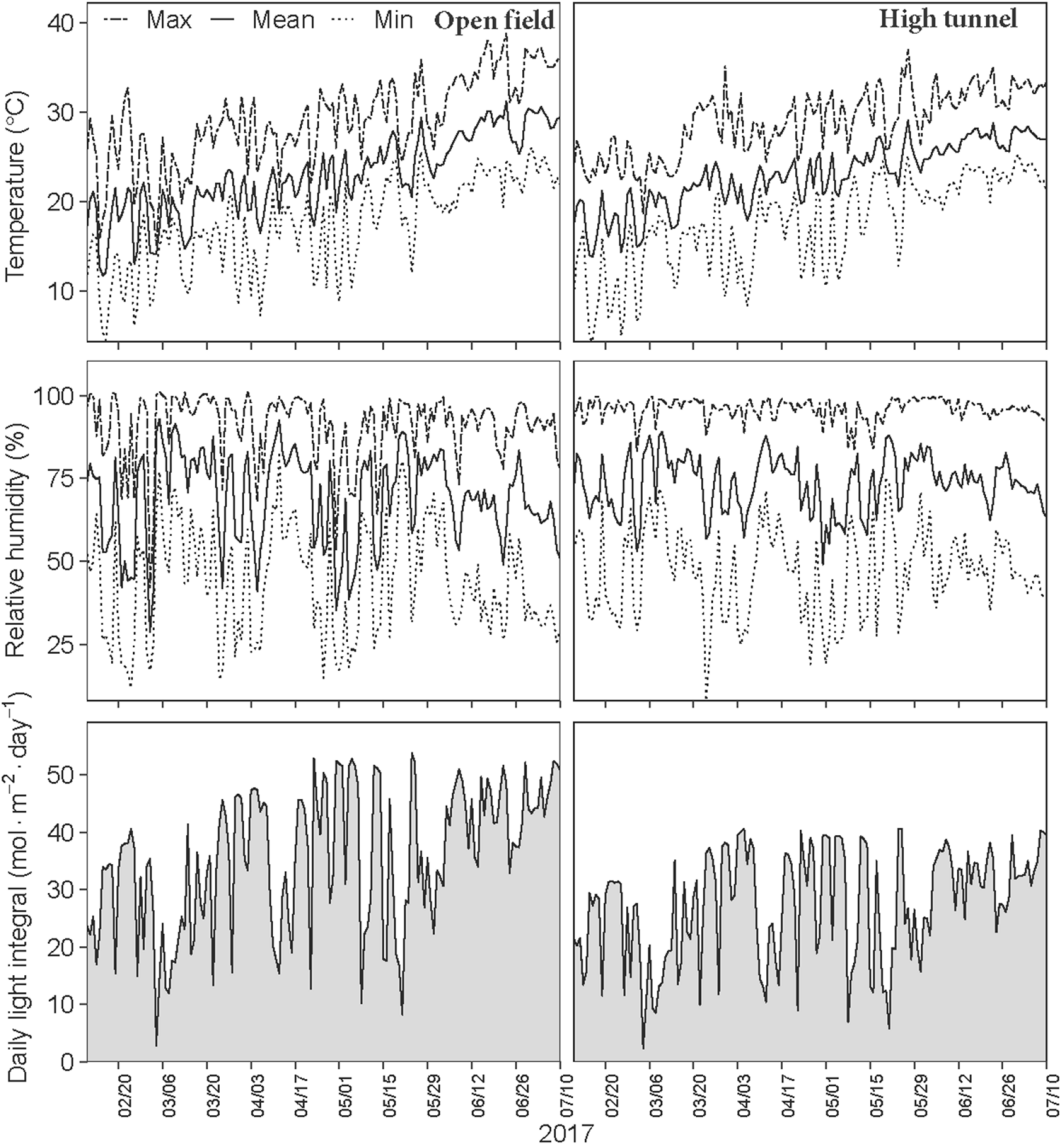
Variations in minimum, mean, and maximum air temperatures, relative humidity, and daily light integral recorded in high tunnel and open field production systems during the 2017 growing spring season in Uvalde, TX.

During the growing season of 2017, the weather parameters especially temperature, relative humidity, and daily light integral were sensibly different between the two production systems (Fig. 1). During the early vegetative growth phase of plants in the high tunnel in the Uvalde location, the daily average air temperatures were about 0.5 to 2.1 °C higher than in the open field (Fig. 1). The average daily relative humidity was 15% higher under the high tunnel compared with the open field condition. In contrast, the average daily light integral (DLI) decreased by 17%, ranging from 11 to 30 mol·m^−2^·d^−1^ in the high tunnel versus 15 to 40 mol·m^−2^·d^−1^ in the open field. During the transplant establishment in the open-field where transplanting occurred on 26 Mar. 2017, the average daily values of temperature, RH, and DLI were 20.6 °C, 65.7%, 40.5 mol·m^−2^·d^−1^, respectively. Environmental conditions prevailing during the transplanting in the field were thus warmer, hastening the growth cycle (116 d) in the open-field compared with high tunnel (150 d).

During the reproductive development, especially from beginning of flowering to first harvest (41–84 DAT) from mid-April to mid-June under the high tunnel, the daily average maximum temperatures ranged from 24.4 to 35.1 °C compared with 21.6 to 33.1 in the open-field. From the first to final harvest (84–150 DAT), the daily average maximum temperatures were between 25.4 to 37 °C. From flowering to first harvest in the open-field trial (32–75 DAT), the range of maximum daily temperature recorded (between late April to early June) was from 24.5 to 35.9 °C. Higher maximum daily temperatures (up to 38.8 °C) were recorded thereafter until the final harvest in early July.

Similar to the reduction in DLI observed under the high tunnel during the vegetative growth stage, the average DLI during the reproductive development were also consistently lower under the high tunnel, ranging from 5.8 to 40.6 mol·m^−2^·d^−1^ compared with 8.1 to 53.8 mol·m^−2^·d^−1^ under open-field condition. Average relative humidity was within similar ranges under both production systems.

In addition to microclimate modification, high tunnels also shelter plants from precipitation compared with the open-field plots which received 217 mm total seasonal rainfall during the 2017 growing season (data not shown). The rainfall during the 2017 season was more concentrated from transplanting to early fruit set which received 95% of the total seasonal rainfall.

### Yield traits in 2017 season

During the 2017 growing season in the Uvalde location, total and marketable yields were influenced by a significant interaction between production system (HT versus OF) and graft combination. Cultivation under HT conditions increased total yield by 116 to 167% relative to OF (Table 2). Higher marketable yield in HT compared with OF was due to significantly higher number of fruits per plant (54 in HT vs 26 in OF) and average fruit weight.

**Table 2:** Yield and yield components of grafted and non-grafted tomato grown in high tunnel and open field during the spring season of 2017 at Uvalde, TX.

Treatment		Yield (Mg ha^−1^)			Avg. marketable fruit weight (g fruit^−1^)	Marketable fruit count (no./plant)	Marketable fruit by size class (%)^1^		
		Total		Marketable			Extra-large	Large	Medium
Production system									
High tunnel (HT)		133.1	–	128.5	225	54	35.8	31.4	32.8
Open field (OF)		–	54.9	52.8	197	26	27.4	29.2	43.4
Graft									
		HT	OF						
TAM		108.8 b	46.2 b	73.1	171 c	39 ab	15.6 d	31.2	53.2 a
TAM/ES		121.2 ab	56.6 ab	86.0	176 c	44 a	21.7 cd	28.8	49.5 ab
TAM/MU		132.2 ab	57.6 ab	91.0	188 c	44 a	25.0 c	31.0	44.0 b
TY		138.6 ab	52.4 ab	91.8	229 b	37 b	36.3 b	31.5	32.2 c
TY/ES		148.2 a	57.4 ab	99.7	240 b	38 b	42.9 ab	30.2	26.9 cd
TY/MU		149.8 a	59.0 a	102.2	262 a	36 b	48.3 a	29.3	22.4 d
**Factor**	**df**		P > F						
Production system (P)	1	< .0001		< .0001	0.0011	< .0001	0.0027	0.1552	0.0013
Graft (G)	5	0.0004		0.0002	< .0001	0.0138	< .0001	0.7121	< .0001
P x G	5	0.0486		0.0459	0.3821	0.4865	0.3242	0.0002	0.0001

Mean separation by Tukey’s honestly significant difference test at P < 0.05.

^1^ Marketable fruit were graded according to the U.S. commercial trade standards.

Within each production system, grafting with rootstock improved both total and marketable yields but the extent of the increase varied with the scion. In the high tunnel production system, total yields of ‘TAMU Hot Ty’ grafted plants onto ‘Estamino’ (TAM/ES) and ‘Multifort’ (TAM/MU) were higher by 11 and 21% than that of the non-grafted scions ‘TAMU Hot Ty’, respectively. With the scion ‘Tycoon’ (TY), these increases were 6 and 8% for ES and MU rootstocks, respectively. In the open field (OF) system, ‘Estamino’ and ‘Multifort’ increased total yield of ‘TAM’ by 23 and 25%, respectively while increasing those of TY by 10 and 13%. Similar trends were also observed for the marketable yields with less increase in ‘Tycoon’ than ‘TAM’. Higher yield of ‘TAM Hot Ty’ grafted plants was due to both higher average fruit weight and marketable fruit count. On average, there were 14% more marketable fruit count per plant of grafted ‘TAM Hot Ty’ than the non-grafted plants while average fruit weight was 7% higher in these grafted plants compared with the non-grafted plants. For the scion ‘Tycoon’, the increased yield of grafted plants was essentially related to higher average fruit weight, particularly in ‘TY/MU’ with 14% greater fruit weight than those of the non-grafted TY.

Distribution of marketable fruit size was also greatly affected by both the production system and grafting with rootstock (Table 2). There were more fruits of extra-large size in HT than in OF while higher percentage of medium fruit was harvested in OF. Moreover, grafting with rootstocks also led to more extra-large fruits. For example, the percentages of extra-large fruit of ‘TY/ES’ and ‘TY/MU’ were higher than those of non-grafted plants ‘TY’ while similar proportions of large fruit were observed among the three types of plants.

### Combined analysis of variance of yield traits in 2018 season

The pooled analysis of variance on the multi-environment trials showed significant effects of environment (location, production system), genotype, and genotype by environment (G × E) interaction for total and marketable yields, marketable fruit number per plant and average marketable fruit weight (Table 3). Partitioning of the total variance as percentage of total sum of squares indicated higher percentage of variation due to environment for all these traits ranging from 71 to 86%. Within the environment, a larger proportion of the variation was due to production system (P) than location for total and marketable yields, and marketable fruit count while greater variation of environment for average fruit weight was due to location. More specifically, of the 85% E variation in total yield, 46% was attributable to P, 17% to location (L), and 36% to production by location interaction (L × P). Grafting had weaker effects on these traits, accounting for 4.81% of total variation in total yield and 10.81% of total variation in average marketable fruit weight. The G × E interaction accounted for 4.29%, 4.37%, 4.64%, and 6.69% of total sum of square for total yield, marketable yield, marketable fruit count per plant, and average marketable fruit weight, respectively (Table 3).

**Table 3:** Significance values and variance components (presented as percentages of the total sum of squares) for yield and yield components of six grafted and non-grafted tomato treatments evaluated in two production systems and four locations in Texas.

Source	DF	Total yield		Marketable yield		Mark. fruit count		Avg. marketable fruit wt	
		MS	%ESS	MS	%ESS	MS	%ESS	MS	%ESS
Environment (E)	7	27,513***	85.26	24,657***	84.54	5,856***	86.14	39,992***	71.22
Location (L)	3	11,229***	17.50	11,904***	20.69	3,150***	23.04	34,793***	37.28
Production system (P)	1	89,177***	46.30	76,673***	44.42	13,677***	33.35	29,008***	10.36
L × P	3	23,242***	36.20	20,071***	34.88	5,960***	43.60	48,852***	52.35
Replication within E	16	130^ ns^	0.92	128^ ns^	1.00	44^ ns^	1.49	748^ ns^	3.04
Graft (G)	5	2 173***	4.81	2,220***	5.44	147***	1.55	8,497***	10.81
G × E	35	277**	4.29	255***	4.37	63*	4.64	751**	6.69
G × L	15	343**	2.28	277**	2.04	73*	2.30	836*	3.19
G × P	5	163^ ns^	0.36	122^ ns^	0.30	43^ ns^	0.46	335^ ns^	0.43
G × L × P	15	248**	1.65	277**	2.04	59*	1.88	804*	3.07
Pooled Error	80	133	4.72	118	5.84	37	6.18	405	8.24

DF = Degrees of freedom, MS = Mean Square, %ESS = % Explained Sum of Squares.

ns, *, **, ***Nonsignificant or significant at P < 0.05, 0.01, or 0.001, respectively.

### Polygon view of GGE biplot (“which-won-where” pattern)

**Figure 2:**
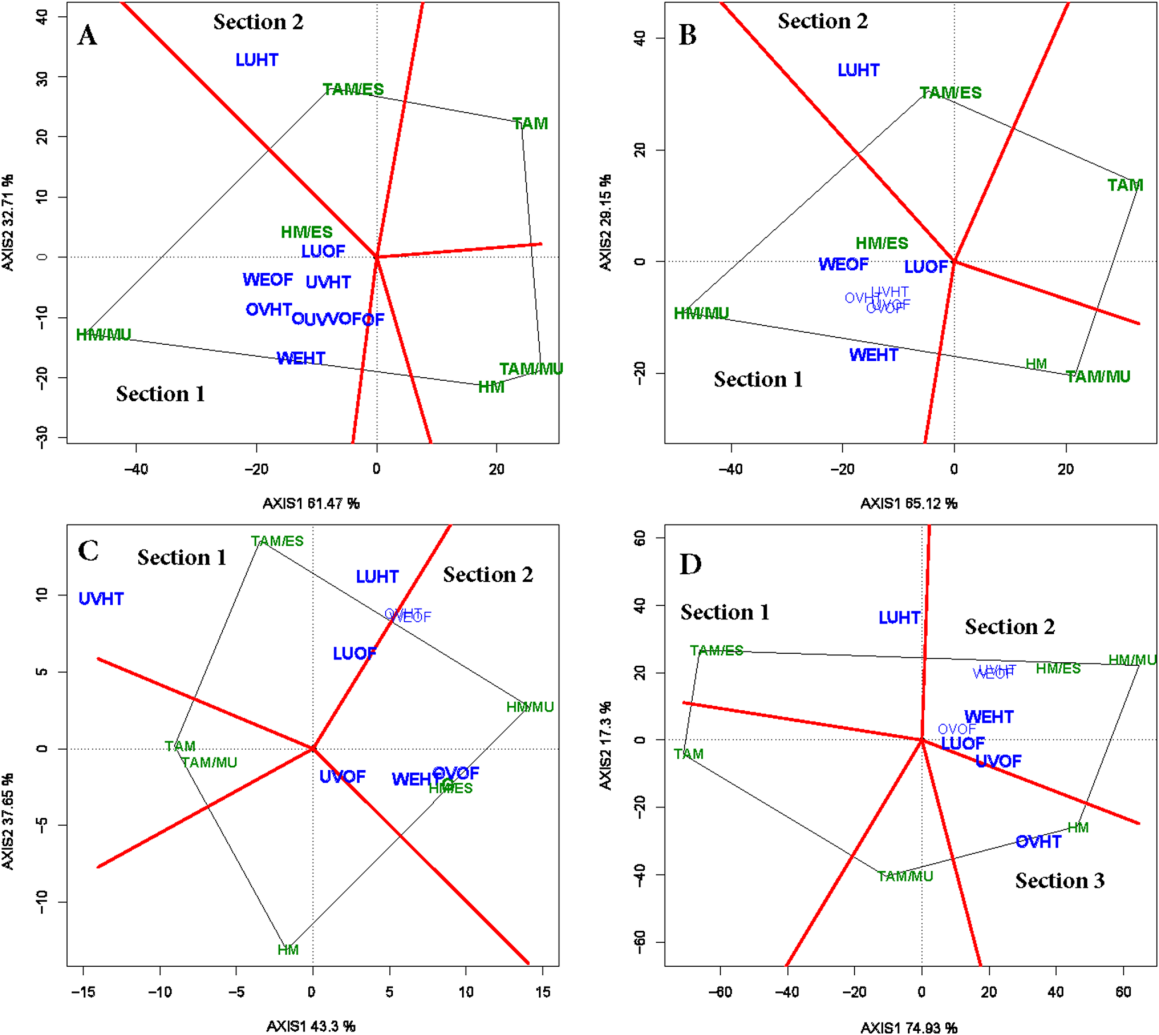
The polygon (which-won-where) view of genotype main effects plus genotypic × environment interaction effect (GGE) biplot of six grafted tomato treatments tested in 4 locations and 2 production systems for total (**A**) and marketable (**B**) fruit yields, marketable fruit count (**C**), and average marketable fruit weight (**D**). The biplots were based on Scaling = 0, Centering = 0, and singular value partitioning (SVP = 2).

In G × E analysis, the polygon view of the GGE-biplot is often used to explicitly visualize the performance of tested genotypes within environment (“which-won-where” pattern) and to divide the environments into potential mega-environments. The polygon is constructed by connecting extreme genotypes of the biplot in such a way that all tested genotypes are included in the polygon. Perpendicular lines from the biplot origin to the sides of the polygon divide the biplot into sectors of environments. In the 2018 multi-location trial, the environmental markers fell into two or three sectors for each of the traits, thus dividing the 8 environments into 2 or 3 mega-environments. For total and marketable yields, the first mega-environment encompassed LU-OF, OV-HT, OV-OF, UV-HT, UV-OF, WE-HT, and WE-OF while LU-HT represented the second mega-environment (Fig. 2A,B). For marketable fruit count (Fig. 2), LU-HT, LU-OF, UV-HT, OV-HT, and WE-OF were all included in the first mega environment while UV-OF, OV-OF, and WE-HT formed the second mega-environment. Three mega-environments could be identified for average fruit weight (Fig. 2D).

The vertex genotype in each sector represents the winning genotype in that environment or set of environments. As such, HM/MU gave the highest yield and was the best performing genotype in environments sharing a common mega-environment, whereas TAM/ES was the winning genotype in sector 2. In contrast, HM, TAM, and TAM/MU represented vertex genotype in a sector with no association with any of the environments and thus performed poorly in all test environments. Similarly, HM/ES fell within the polygon for yields and appeared less responsive to the test environment than the vertex genotypes. For fruit count per plant, HM/MU, TAM/ES were the winning graft combinations for each of the two mega-environments, respectively. These two graft combinations were also the respective winning genotypes for average fruit weight along with HM in the sector representing the environment OV-HT.

### Genotype evaluation based on GGE biplots

Given the significant G × E effects on the yield traits, genotypes should be evaluated in terms of mean trait performance and stability across testing environments.

#### Genotype means comparison

Total yield ranged from 73.03 to 98.19 Mg ha^−1^ with an average of 81.96 Mg ha^−1^. Similarly, marketable yield varied from 61.93 to 88.43 Mg ha^−1^ with an average of 73.14 Mg ha^−1^ (Table 4). HM/MU had the highest total and marketable yields while yields of non-grafted HM, TAM and grafted TAM/MU were lower than the respective grand mean. Greater marketable yields exhibited by the HM/MU combination was attributable to both higher marketable fruit count per plant (40) and higher average marketable fruit weight (215 g frt^−1^). Respective values for non-grafted HM were 33.9 and 205.16 which are both lower than the grand average (Table 4). Similarly, higher marketable yield in TAM/ES compared to non-grafted TAM was related to greater yield components.

**Table 4:** Means (corrected by least squares) for total and marketable yields, marketable fruit count per plant, and average fruit weight of six grafted and non-grafted tomatoes tested in eight environments in Texas.

Graft	Total yield (Mg ha^−1^)	Marketable yield (Mg ha^−1^)	Fruit count (No./plant)	Fruit weight (g)
HM	77.9 bc	70.1 cd	34 c	205.1 a
HM/ES	86.6 b	78.4 b	36 abc	208.2 a
HM/MU	98.2 a	88.4 a	41 a	215.1 a
TAM	73.0 c	61.9 d	36 bc	169.6 b
TAM/ES	82.4 bc	73.2 bc	39 ab	175.8 b
TAM/MU	73.5 c	66.1 cd	36 abc	185.2 b
Grand mean (n = 24)	81.9	73.1	37	193.2
CV %	14.1	14.8	16	10.4

* Mean separation by Tukey’s honestly significant difference test at P < 0.05.

CV: coefficient of variation.

#### Univariate stability statistics methods

In this study, univariate statistic including regression coefficient (${b}_{i}$), deviation from regression (${S}_{d}^{2}$) and Shukla stability variance (${\sigma }_{i}^{2}$) were used to evaluate genotype stability performance across test environments. Typically, a stable genotype should have high mean performance, regression coefficient ${(b}_{i})$ near 1, a low deviation from regression (${S}_{d}^{2}$), and with a low value of Shukla’s stability variance. Resultant ${b}_{i}$ values ranged from 0.49 (HM/MU) to 2.05 (TAM/ES) for total yield, 0.55 (TAM) to 1.53 (TAM/ES) for marketable yield, 0.68 (HM/MU) to 2.20 (TAM/MU) for fruit count per plant, and from 0.49 (TAM) to 2.28 (HM) for average fruit weight (Table 5). As such, HM/ES with the second highest total yield and a ${b}_{i}$ value of 0.86 was the most stable over different environments. While for marketable yield, HM/MU with the highest yield and ${b}_{i}$ 0.81 represented the most stable genotype. For marketable fruit count, TAM/MU with ${b}_{i}$ (2.20) appeared to be the least stable. Based on the range in values of ${S}_{d}^{2}$, HM/ES represented the most stable for total and marketable yield, while HM/MU for fruit count per plant and average fruit weight. Considering all these univariate statistics, ranking genotypes from the most to the least stable for total yield was as follows: HM/ES > HM/MU > TAM > TAM/MU > HM > TAM/ES.

**Table 5:** Means (corrected by least squares) (M), regression coefficient (${b}_{i}$), deviation from regression (${S}_{d}^{2}$), and Shukla’s stability variance (${\sigma }_{i}^{2}$) for total and marketable yields, marketable fruit count, and average fruit weight of 6 grafted and non-grafted tomatoes tested in two production systems and four locations in Texas.

Trait	Genotype	M	${b}_{i}$	${S}_{d}^{2}$	${\sigma }_{i}^{2}$
Total yield	HM	77.07	1.10	191.23	355.64*
	HM/ES	86.63	0.86	38.26	7.94***
	HM/MU	99.06	0.49	80.97	54.72
	TAM	73.03	0.68	145.59	277.58
	TAM/ES	82.45	2.05	368.48	536.82***
	TAM/MU	73.52	0.79	221.35	333.94*
Marketable yield	HM	68.84	1.47	159.08	229.41
	HM/ES	78.43	0.59	51.76	19.57
	HM/MU	89.89	0.81	82.35	65.78
	TAM	61.92	0.55	157.47*	170.02
	TAM/ES	73.26	1.53	415.97*	592.14***
	TAM/MU	66.12	1.05	232.92*	328.58*
Fruit count per plant	HM	33.90	0.93	42.51	56.28
	HM/ES	36.51	0.75	32.81	50.03
	HM/MU	40.18	0.68	19.63	75.49
	TAM	35.58	0.94	31.67	57.86
	TAM/ES	39.78	0.93	44.14	77.67
	TAM/MU	35.98	2.20*	47.93	61.07
Avg. fruit weight	HM	205.15	2.28*	1,011.45*	1,159.29*
	HM/ES	208.20	0.74	502.53	141.53
	HM/MU	215.16	0.98	275.60	420.68
	TAM	169.64	0.49	356.01	241.58
	TAM/ES	175.84	0.65	1,012.59*	1706.73***
	TAM/MU	185.24	1.44	641.03	735.80

* Significantly different from unity for the regression coefficients (${b}_{i}$) and from zero for the deviation from regression (${S}_{d}^{2}$) at 0.05 level of probability.

*, **, and *** Significantly larger than the within-environment variance (${\sigma }_{e}^{2})$ at 0.05, 0.01, and 0.001 levels of probability, respectively.

When these stability parameters are associated with low trait mean performance, genotypes in question are poorly adapted to all environments. As such, for marketable yield, TAM/MU with ${b}_{i}$ of 1.05 but the second lowest marketable yield could be considered as a poor performer but stable genotype.

#### Mean vs. stability and genotype comparison with ideal genotype views of GGE biplot

**Figure 3:**
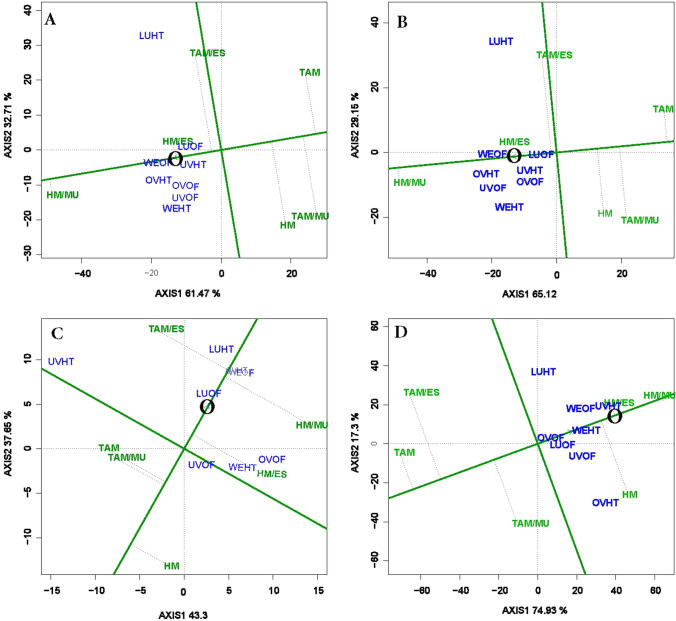
The mean vs. stability view of genotype main effects plus genotypic x environment interaction effect (GGE) biplot of six grafted tomato treatments tested in 4 locations and 2 production systems for total (**A**) and marketable (**B**) fruit yields, marketable fruit count (**C**), and average marketable fruit weight (**D**). The biplots were based on Scaling = 0, Centering = 2, and SVP = 1.

The average environment coordinate (AEC) view of the GGE biplot allows the visualization of genotype mean performance for specific trait and the stability of genotypes across environments (Fig. 3). The horizontal line represents the AEC abscissa, with the arrow pointing to the greatest genotype main effect across environments (left lower end). Therefore, HM/MU and HM/ES exhibited the highest mean total and marketable yields while TAM, the lowest yields (Fig. 3A,B, right upper end). Similarly, TAM/ES and HM/MU had the highest marketable fruit count and average marketable fruit weight, respectively (Fig. 3C,D).

The perpendicular line to the abscissa represents the AEC ordinate and indicates the level of stability of each tested genotype. The most stable genotype is usually positioned on the AEC abscissa (shortest projection on the abscissa). As such, TAM/ES was highly unstable whereas HM/MU was highly stable followed by HM/ES for total and marketable yields (Fig. 3A,B). Similarly, HM/MU and HM/ES also demonstrated the highest stability for average marketable fruit weight while TAM/ES and HM the least stable (Fig. 3D). For marketable fruit count, non-grafted HM and TAM seemed the most stable in contrast to HM/MU or TAM/ES (Fig. 3C).

**Figure 4:**
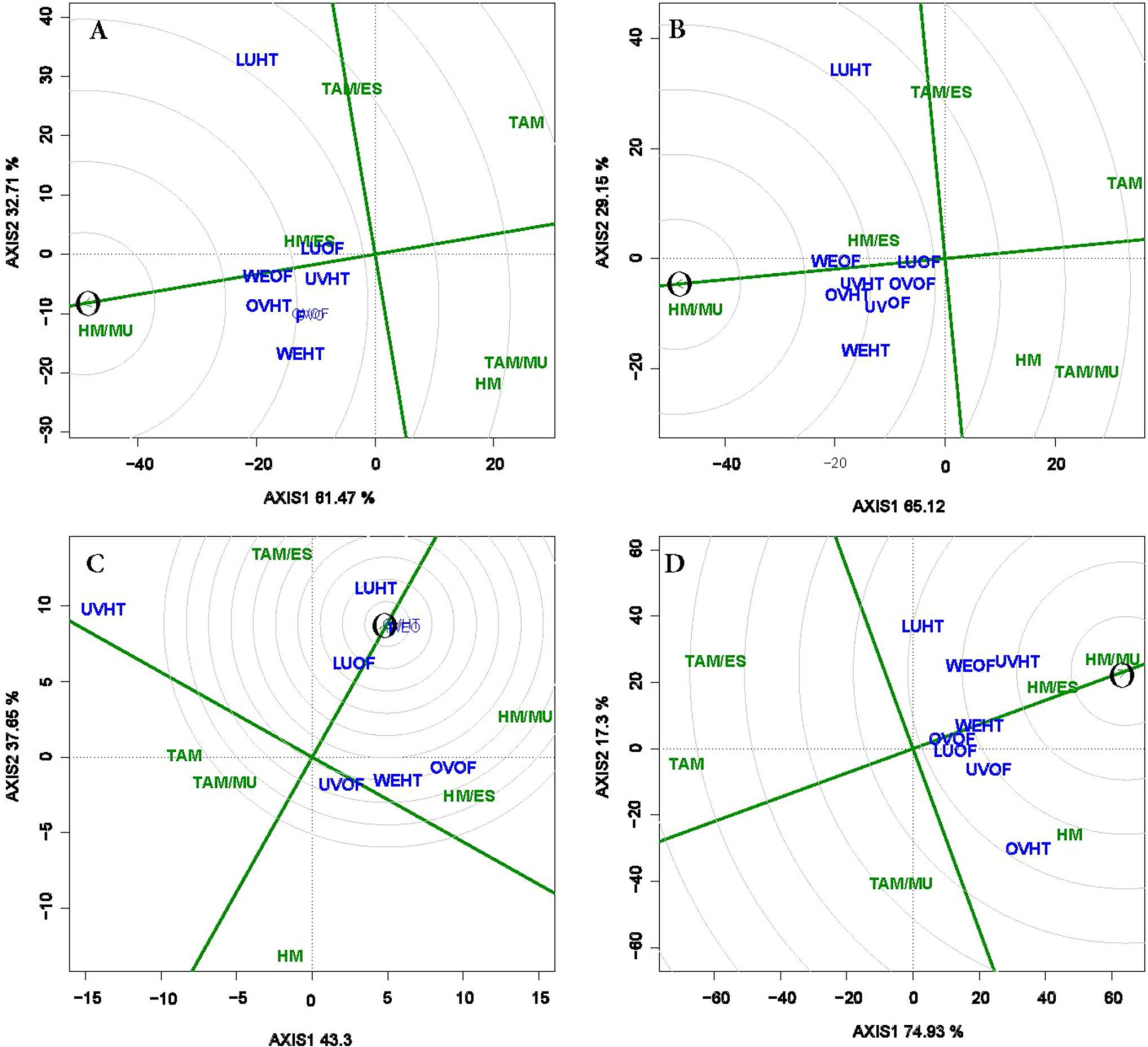
The genotypes comparison with ideal genotype view of genotype main effects plus genotypic × environment interaction effect (GGE) biplot of six grafted tomato treatments tested in 4 locations and 2 production systems for total (**A**) and marketable (**B**) fruit yields, marketable fruit count (**C**), and average marketable fruit weight (**D**). The biplots were based on Scaling = 0, Centering = 2, and SVP = 1.

In addition, the “mean vs. stability” view also reveals the “ideal” genotype which in principle should be characterized by both high mean yield and high stability. In general, any genotype located near the arrow on the AEC abscissa often indicates this ideal genotype. In this study, for total and marketable yields, HM/ES could be viewed as the ideal genotype given its closeness to the circle (Fig. 3A,B). With the identification of the “ideal” genotype, all genotypes can also be compared with this ideal genotype. The GGE biplot view depicting genotypes comparison with the “ideal” genotype encompasses concentric circles with the genotype or group of genotypes falling within the most inner circle represent the most desirable genotype (Fig. 4). In this study, the biplot showed HM/MU within this inner circle at the head of the arrow for total yield, marketable yield and average marketable fruit weight (Fig. 4A,B,D). Thus, HM/MU represented the most desirable genotype for each of these traits. In contrast, for marketable fruit count, no genotypes were found in the inner circle (Fig. 4C). TAM/ES which was located next to the ideal circle could be considered as the most desirable genotype for this trait.

### Environment evaluation based on GGE biplots

Similar to genotype evaluation, testing environments can also be evaluated to identify environments that are most performing and also the ones that reveal more information about differences among grafted and non-grafted genotypes (discriminating ability of the environments and ranking of genotypes).

#### Environment means comparison

Averaged over the six genotypes, the mean comparison of environments showed that protected structure (HT) significantly increased fruit yields compared with open-field (OF) production system (Table 6). Marketable yields in HT were 8–345% higher than those in OF across all four locations. LU-OF had the lowest yields while HT in Lubbock (LU-HT) and in Uvalde (UV-HT) exhibited the highest yields. Highest marketable yields in LU-HT were related to both higher marketable fruit count and average fruit weight while the increase in UV-HT was mainly due to greater fruit count.

**Table 6:** Environmental means (corrected by least squares) for total and marketable yields, marketable fruit count, and fruit size of six grafted and non-grafted tomatoes tested in eight environments.

Environment	Genotype						
	HM	HM/ES	HM/MU	TAM	TAM/ES	TAM/MU	Grand mean
	Total yield (Mg ha^−1^)						
LU-HT	109.3 b	144.7 a	149.3 a	143.3 a	158.7 a	103.7 b	134.8
LU-OF	39.3 ab	46.3 ab	52.0 a	35.6 ab	46.7 ab	34.7 b	42.4
OV-HT	72.3 b	78.0 ab	100.0 a	57.7 b	76.0 b	67.3 b	75.2
OV-OF	68.0 a	78.3 a	82.7 a	57.0 a	61.0 a	65.3 a	68.7
UV-HT	146.1 b	151.7 ab	164.8 a	147.1 b	147.4 ab	149.0 ab	151.0
UV-OF	67.1 a	56.5 ab	65.7 a	47.5 b	48.7 b	49.6 b	55.8
WE-HT	70.7 b	71.3 b	87.8 a	53.7 c	52.9 c	61.3 bc	66.3
WE-OF	50.6 bc	66.2 ab	83.1 a	42.2 c	68.2 ab	57.2 bc	61.3
	Marketable yield (Mg ha^−1^)						
LU-HT	88.0 cd	119.3 ab	122.0 ab	107.6 bc	134.0 a	78.7 d	108.3
LU-OF	22.3 ab	25.0 ab	31.3 a	20.0 b	24.7 ab	21.7 b	24.2
OV-HT	67.6 b	70.0 ab	89.3 a	51.3 b	67.3 b	62.0 b	67.9
OV-OF	61.0 a	73.7 a	76.0 a	49.7 a	54.0 a	58.0 a	62.1
UV-HT	139.7 b	149.5 ab	161. 3 a	136.0 b	140.8 b	143.7 ab	145.2
UV-OF	66.3 a	56.0 ab	65.3 a	46.7 b	48.4 b	49.3 b	55.3
WE-HT	68.4 b	69.4 b	85.2 a	47.9 c	50.5 c	59.6 bc	63.5
WE-OF	47.8 bc	64.6 ab	81.6 a	36.1 c	66.4 ab	56.0 bc	58.8
	Fruit count (No./plant)						
LU-HT	41.0 b	51.0 ab	51.0 ab	50.0 ab	56.0 a	42.0 b	49.0
LU-OF	19.0 c	21.0 bc	29.0 a	27.0 ab	26. abc	22. 0 bc	24.0
OV-HT	35.0 a	40.0 a	42.0 a	31.0 a	33.0 a	34.0 a	36.0
OV-OF	25.0 a	31.0 a	35.0 a	26.0 a	36.0 a	27.0 a	30.0
UV-HT	71.0 abc	69.0 c	70.0 bc	81.0 abc	85.0 a	84.0 ab	77.0
UV-OF	27.0 a	22.0 ab	26.0 a	23.0 ab	24.0 ab	20.0 b	24.0
WE-HT	31.0 ab	32.0 ab	37.0 a	27.0 b	28.0 b	31.0 ab	31.0
WE-OF	20.0 b	26.0 b	31.0 a	19.0 b	30.0 a	27.0 ab	25.0
	Fruit weight (g)						
LU-HT	197.7 ab	217. 0 a	222.0 a	200.3 ab	224.3 a	174.0 b	206.0
LU-OF	109.6 a	107.0 a	98.3 a	69.3 b	89.0ab	93.0 a	94.0
OV-HT	289.0 ab	259.0 abc	309.3 a	220.3 bc	209.0 c	266.3 abc	259.0
OV-OF	194.0 a	204.6 a	201.0 a	178.0 a	176.0 a	187.3 a	190.0
UV-HT	181.2 bc	204.3 ab	215.5 a	156.7 cd	153.4 d	160.5 cd	179.0
UV-OF	225.6 a	246.9 a	238.0 a	191.8 b	184.5 b	230.2 a	219.5
WE-HT	199.2 ab	200.5 ab	211.5 a	163.3 c	166.5 c	176.2 bc	186.0
WE-OF	224.6 ab	226.1 ab	246.1 a	177.3 d	203.9 cd	194.3 cd	212.1

* Means followed by the same lowercase letter within a row are not significantly different at P < 0.05 based on Tukey’s honestly test.

#### Discriminating ability of test environments

**Figure 5:**
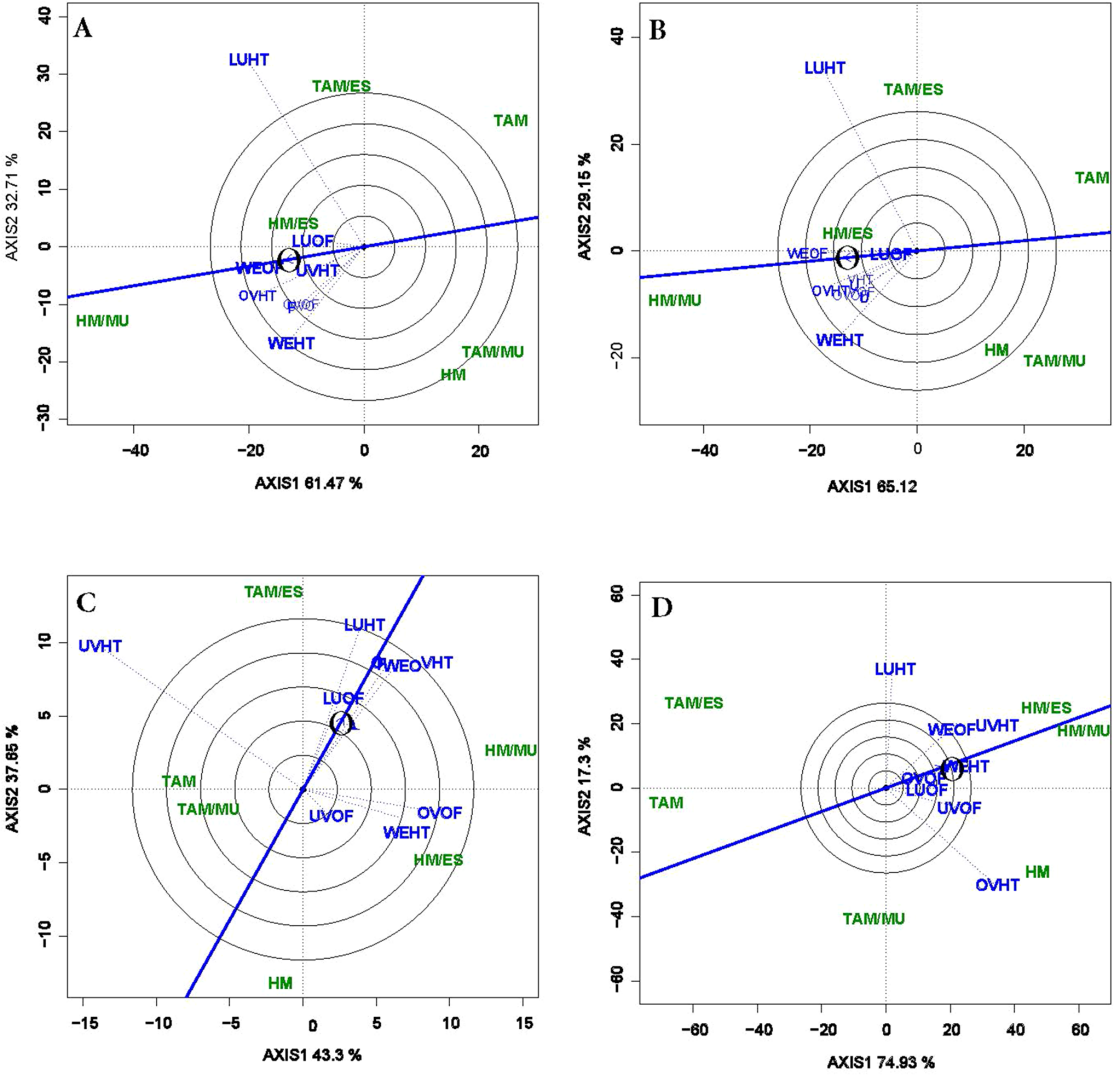
The ‘discriminating power’ view of genotype main effects plus genotypic x environment interaction effect (GGE) biplot of six grafted tomato treatments tested in 4 locations and 2 production systems for total (**A**) and marketable (**B**) fruit yields, marketable fruit count (**C**), and average marketable fruit weight (**D**). The biplots were based on Scaling = 0, Centering = 2, and SVP = 1.

In the discriminating power view of the GGE biplot, the extent of the ability of each testing environment to discriminate among genotypes is depicted by the length of the environment vector line between the marker of the environment and the biplot origin; the longer the vector, the higher the discriminating ability. Therefore, the environment (LU-HT) with the longest length seemed the most discriminating of the genotypes, which means it revealed more information about differences among the tested genotypes in terms of total and marketable yields. In contrast, the environments LU-OF and UV-HT with the shortest length were the least discriminating, suggesting the genotypes performed quite similarly in each of these environments for total and marketable yields (Fig. 5). Based on length of the environment vectors, ranking the 8 environments in terms of their ability to discriminate genotypes was as follows: LU-HT > WE-HT > OV-HT = WE-OF > OV-OF = UV-OF > UV-HT > LU-OF respectively (Fig. 5A). For example, in LU-HT (the most discriminating environment), marketable yields ranged from 79 to 134 Mg ha^−1^ whereas in the least discriminating environment (LU-OF), the yields ranged from 30 to 31 Mg ha^−1^. For the marketable fruit count, UV-HT and UV-OF represented the most and the least discriminating environments, respectively (Fig. 5C). LU-HT and OV-HT are more discriminating environments for the average marketable fruit weight (Fig. 5D).

## Discussion

Variation in genotype performance across locations is often related to changes in environmental factors such as temperature, relative humidity, rainfall amount and distribution, light intensity, photoperiod, soil moisture, and cultural practices^12^. In this study, greatest proportion of total variation in the genotypes (grafted treatments) performance was attributed to environment (E), whereas genotype (G) and G × E sources of variation were relatively smaller. Dia et al.^3^ found similar observations in yield traits of watermelon genotypes evaluated across different environments in U.S. Our trials indicated that a larger source of E variation in grafted tomato yield performance was due to production system than location. On the one hand, presence of significant G × E for all the yield traits indicated that grafted treatments responded differently across the testing environments. This significant G × E was further confirmed by the polygon views of the GGE biplot which showed there were two to three mega-environments with specific winning genotypes in each different mega-environment. In addition, based on the relative performance of the genotypes, there was a difference in the genotype rankings across the testing environments, which also demonstrated the existence of crossover G × E^38^. On the other hand, significant G × E also illustrated the existence of unstable genotypes which often suggests the need to accurately explore the stability in yield traits performance of the genotypes across the testing environments. Such analysis allows the identification of ideal genotypes with highest mean performance and high stability across the environments^39^. Based on mean vs. stability views of the GGE biplot and other stability parameters (${b}_{i}, {S}_{d}^{2}, and {\sigma }_{i}^{2}$), Dia et al.^3^ on watermelon and Ummyiah et al.^17^ on hybrid tomato identified groups of stable and unstable genotypes in terms of yield traits. Similarly, in this study, the grafted treatments could be classified in three groups with respect to these stability parameters. Group 1 encompassed HM/ES and HM/MU which exhibited the highest yield and high stability coefficients (${b}_{i}$ close to unity, low ${\sigma }_{i}^{2}$). HM/ES and HM/MU were also characterized by higher and stable yield attributes (especially average fruit weight). Ortiz and Izquierdo^18^ found similar observation with a yield stability study on tomato genotypes. As such, these two grafted treatments appeared more adapted to the testing environments and could be more desirable for consideration for enhancing tomato productivity across the locations in Texas. TAM/ES fell in the group 2, with high yields (third highest yield) but highly unstable (${b}_{i}$ greater than 1) and thus seemed more suitable for specific environment (LU-HT). While TAM/MU, TAM and HM could be grouped together (group 3) with yield lower than the grand mean but were equally stable. This indicates that these three genotypes are poorly adapted to all environments.

Across all four locations, high tunnel cultivation had significantly higher yields than open-field cultivation, and this is consistent with the documented benefits of season extension and protection of crops from sun, wind, excessive rainfall in high tunnel production systems^40^. The recorded increase in marketable yields ranged from 8% in Overton and Weslaco, to 347% in Lubbock. These results were similar to previous findings on comparative performance of tomato in high tunnel vs. field production^41–43^, strawberry^44^, lettuce^45,46^, and specialty cut flowers^47^. Highest yield benefits of HT over OF were registered in Uvalde and Lubbock. This yield advantage in HT over OF could be primarily related to relatively favorable microclimate inside HT. Particularly at Uvalde location, HT allowed up to six weeks of early-season extension over OF. With the early transplanting, plants grown under the HT benefited from relatively more clement microclimate (especially mild air temperatures) which could have stimulated early vegetative growth and flower development, thereby, resulting in early and more harvests in HT over OF system. Due to risk of late frost, transplanting in OF occurred late March. As a result, high day and night average temperatures (sometimes higher than 32 °C and 21 °C, respectively) prevailed during the active reproductive development of growth cycle. These episodes of high temperature could have resulted in heat stress damage including production and release of fewer pollen grain^48^ potentially leading to poor fruit set and low yield^49^. Higher temperature in combination with heavy rain events and wind in the OF system could in part explain lower yields achieved in OF compared with HT. Similarly, these environmental stresses, especially high temperatures and wind, likely contributed to the lowest yields recorded in open-field trial in Lubbock where frequently high winds and dust are common during the spring. Although weather parameters were not monitored during this study in Lubbock, Wallace et al.^45^ previously measured wind speed 46 times greater in the OF (19.8 km·h^−1^) compared with the HT. At Uvalde location, values of DLI were consistently lower in HT compared with OF. Kitta et al.^50^ observed similar findings under screenhouse condition compared with that of OF. Yet, these authors measured substantially higher light use efficiency on sweet pepper plants in the screenhouse conditions compared to OF. Similarly, Xu^51^ measured 46 to 51% higher photosynthetic production at whole canopy level in raspberries plants grown in HT compared to OF plants, even though single leaf photosynthetic rates were lower in HT plants. Therefore, the yield benefit in HT over OF in this study could also be related to enhanced photosynthetic activities under the HT environment. Unlike in Uvalde and Lubbock, the beneficial effect of using HT is somewhat moderate in Overton and Weslaco locations. Transplanting occurred within the same period (early spring) in both environments in these locations. As such, for protected structures like high tunnels to be advantageous for growing tomato in regions like south Texas, transplanting should occur earlier during cooler weather, which could potentially ensure premium prices associated with early harvest.

Except for a decrease in TAM/MU marketable yield compared to TAM in LU-HT, grafted with either rootstock consistently increased marketable yields by 5 to 69%. This range of percentage increase is similar to that (5 to 80%) reported by Bie et al.^24^ when comparing non-grafted or self-grafted tomato plants. Evidence suggests that yield benefits of grafting with specific rootstocks could be related to rootstock-modulated changes in rootstock-scion signaling, especially long-distance transport of phytohormones and small molecules^52^.

Overall, in line with findings by Grieneisen et al.^25^, our study showed that rootstock-modulated changes in yield traits are also dependent on the scion and testing environment; which clearly explained the significant E, G, and G × E effects for yields and its components. The GGE biplot analysis was effective in displaying the response patterns of these specific effects. It also identified HM/MU and HM/ES as the most stable high-yielding genotypes while TAM/ES as the most unstable. Although a limited number of scion/rootstock combinations were evaluated, this work provides a basis for assessing G × E and stability of yield traits in grafted tomatoes across several production environments. With new rootstocks becoming more available, similar multi-environment trials especially at the regional level are warranted to fully investigate G × E and stability analysis of yield traits in these scion/rootstock combinations.
